# Breeding of new kiwifruit (*Actinidia chinensis*) cultivars with yellow (golden) fleshed and superior characteristics

**DOI:** 10.1186/s12870-024-05768-0

**Published:** 2024-11-05

**Authors:** Mojdeh Asadi, Mahmood Ghasemnezhad, Adel Bakhshipour, Jamalali Olfati, Arif Atak

**Affiliations:** 1 Department of Horticultural Sciences, Faculty of Agricultural Sciences, University of Guilan, Guilan, Iran; 2 Department of Biosystem Engineering, Faculty of Agricultural Sciences, University of Guilan, Guilan, Iran; 3 Department of Horticulture, Faculty of Agriculture, Bursa Uludağ University, Bursa, 16059 Türkiye

**Keywords:** Hybrid genotypes, Fruit size, Quality characteristics, Sensory panel, Cultivar candidate

## Abstract

The most widely cultivated species globally is *Actinidia deliciosa* cv. ‘Hayward’. However, in recent years, consumers have shown greater demand for new varieties with novel flesh colour, flavour and appearance in international markets. To meet these expectations, one breeding study was started in 2016 at the University of Guilan in Iran to develop new kiwifruit cultivars with superior characteristics. In this breeding program, 201 female and 534 male hybrid genotypes were obtained from combinations of six different parents using the controlled cross-breeding technique. The evaluations were carried out over three consecutive stages. In the first stage, 201 female hybrid genotypes and control (‘Golden’) were evaluated based on 25 phenological and pomological traits. In the second stage, hybrids passed the desired threshold values in 7 key attributes were selected. Sensory testing in the next stage evaluated these selected hybrid genotypes more. As a result of the first stage, 30 hybrid genotypes with fruit weight (90 ≥ g) were selected. Then, in the second stage, 21 hybrids that showed acceptable dry matter, hue angle, flesh firmness, soluble solid content, acidity, and ascorbic acid were selected. Finally, 13 hybrid genotypes received high scores in sensory testing, and just two hybrid genotypes represented a 100% of novelty with positive texture. These genotypes were selected as potential cultivar candidates. In future studies, fruit yield, disease/pest resistance, and post-harvest performance trials of the 13 selected candidates will be evaluated at different sites. Thus, the ones that give the best results will be introduced to kiwifruit growers.

**Supplementary Information:**

The online version contains supplementary material available at 10.1186/s12870-024-05768-0.

## Introduction

Kiwifruit is recognized as a nutrient-dense fruit, rich in vitamins A, B, C, E, and K, dietary fiber, and phytochemicals such as carotenoids and flavonoids [1]. These components contribute to its extensive pharmacological properties, including antidiabetic, anti-tumor, anti-inflammatory, antioxidant effects, as well as hypoglycemic and hypolipidemic benefits. Therefore, regular consumption of kiwifruit can enhance digestive health and support cardiovascular wellness [1]. According to FAOSTAT (2022), worldwide annual production of kiwifruit was 4.5. China was the global leader in kiwifruit production, contributing about 2.38 million tonnes annually, and Iran is the world’s fifth largest producer of kiwifruit, with an annual production exceeding 300,000 metric tons [2]. While the majority of this production consists of *A. deliciosa* cv. ‘Hayward’, there is a great demand for golden-fleshed varieties both domestically and internationally [3].

The foundation for the global kiwifruit industry is the green-fleshed *Actinidia deliciosa* cultivar ‘Hayward’, selected in New Zealand [4]. ‘Hayward’ is now grown in all major kiwifruit-exporting countries and provides the bulk of globally traded kiwifruit [5]. The success of Hayward fruit is mainly related to its ability to keep the fruit in cold storage for long periods [6], its flavour and its large fruit. However, in recent consumers have shown increasing interest in Actinidia fruit with golden flesh. New varieties have become available as selections from the wild or breeding programmes, and claimed superior traits that distinguish them from the ‘Hayward’ cultivar. Some of these traits include the time of harvest (early), size (large), taste (sweet) and flesh colour (yellow and red) [7, 8]. These new varieties could expand the market window for individual suppliers and add to consumers’ eating experience. New consumers who were previously uninterested in traditional ‘Hayward’ kiwifruit for various reasons (e.g. high acidity, too much hairiness) may be more interested in new varieties’ exciting new eating experience [9]. It is reported that consumers’ general beliefs, attitudes, perceptions, tastes, appearance and novelty regarding fruits are highly influential on their kiwifruit buying preferences and desires [10]. Appearance of fruit is one of the critical factors attracting consumer interest at the point of purchase and setting expectations of what the fruit might taste like. A recent study showed consumers were willing to buy new kiwifruit cultivars [11].

An increasing number of varieties are being produced due to systematic breeding programs involving controlled, planned, interspecific and interspecific crosses [12–14]. The breeding methods, such as controlled crossing, studies on compatibility within species, mutation breeding and interspecific hybridization used in Ferguson et al. [15] and Oliveira and Fraser [16], as well as factorial mating designs, full-sib breeding and selection strategies described by Marsh et al. [17], along with planned hybridizations and selections from wild populations by Ferguson and Seal [18] are outlined. For many years, the most successful kiwifruit cultivar to emerge from a systematic breeding program was *A. chinensis* ‘Hort16A’, the first yellow-fleshed kiwifruit cultivar to be marketed internationally. Apart from yellow-fleshed cultivars selected from the wild in China, new yellow-fleshed cultivars have also been developed from systematic breeding programs in different countries. These include ‘Dori’, Italy [19], ‘Goldrush’, Korea [20], ‘Haegeum̕, Korea [21], ‘Halla Gold̕, Korea [22], ‘Jecy Gold̕, Korea [23], ‘Sanuki Gold̕, Japan [24], ‘Soreli̕, Italy [25], and ‘Y368̕ New Zealand [26].

Kiwifruit (*Actinidia spp*.) is one of the main export crops from Iran. It is cultivated in the favourable climate around the Caspian Sea. The most commonly grown cultivar is *A. chinensis var. deliciosa* ‘Hayward’, although current interest lies in commercializing new yellow-fleshed and red-fleshed cultivars. The kiwifruit breeding programme in Iran was started by Ebrahimi, who imported ‘Hort16A’ seeds from The United States in 2003. Although pollinating the resulting 5 female vines (named EMF1-5) with 7 male vines (EMM1-7) produced relatively small fruit, all of them showed a promising golden colour in the pericarp region. Then, ‘Hayward’ cultivar was crossed with new male genotypes. The three superior genotypes were selected and introduced as EMF6, EMF7, and EMF8 [3]. Therefore, different breeding studies have been started across the country for kiwifruits with different flesh colours due to their unique and attractive appeal [27].

Therefore, the goal of this study was to evaluate the performance of hybrid genotypes to develop golden kiwifruit cultivars with large fruits, novel taste and flavour that consumers will appreciate more and bring high income to growers compared to other kiwifruit cultivars entering the market.

## Materials and methods

### Experimental condition

The study was carried out at University of Guilan, Rasht, Iran (37°11’50.6” N, 49°38’43.7” E), where the climate is almost Mediterranean, with mild and rainy winters and hot and humid summers. The average monthly temperatures range from 8 to 10 °C in winter to 24–27 °C in summer. The minimum monthly temperatures range from 6 to 8 °C in winter to 21–24 °C in summer. In general, winter is wetter than summer. There are rarely adverse climatic events such as spring frosts, winter freezing, wind damage and hail in the main areas of kiwifruit production.

### Hybridization(cross-breeding) and pollination

In the cross-breeding part of this study, genotypes CK02, CK04 and CK06 belonging to *A. chinensis* were used as pollinator (male) parents. Genotypes CK01, CK03 and CK05 belonging to *A. chinensis* were used as female parents (Table 1).

**Table 1:** Kiwifruit cultivars/genotypes used as parents in cross-breeding studies and their combinations

Species	Combination	Symbol	Ploidy level
*Actinidia chinensis*	CK-02×CK-01	CK2-CK98	4x
*Actinidia chinensis*	CK-04×CK-03	CK99-CK165	4x
*Actinidia chinensis*	CK-06×CK-05	CK165-CK202	4x

The genotypes used in the cross-breeding studies as parents were selected from the elite seedlings obtained from open pollination of the tetraploid ‘Sungold’ (G3), ‘Charm’ (G9), ‘Sweet Green’ (G14) and ‘Jintao’ cultivars. Later, different hybridization(cross-breeding) studies were carried out with these parents to develop new varieties with superior characteristics. In order to compare the fruit characteristics of the hybrid genotypes obtained in the study, *A. chinensis* cv. ‘Golden’, developed from ‘Hort16A’ plant commercially grown in northern provinces of Iran, was used as control. The ‘Hort16A’ variety belongs to the *A. chinensis* species and was produced and sold by Zespri under the trade name ‘Gold Kiwifruit’ in the early 2000s.

### Germination

In the winter of 2017, seeds were stratified (5 weeks at 4 °C) in Petri dishes, transferred to an incubator with alternating temperature and light at 24 °C (16 h)/5°C (8 h) and then transferred to a growth chamber with constant temperature (20 °C) and continuous light for germination to occur. After germination, seedlings were transferred to pots filled with sterile medium (peat moss and perlite, 1:1) and kept at greenhouse conditions (80–90% relative humidity, 25–30 °C temperature with supplemental light) for eight months [28].

### Planting and growing of new commercial seedling populations

All 735 (201 females and 534 males) seedlings from Hybridization were planted out in the nursery with high density following instructions recommended by Morgan [29], Testolin and McNellage [30] at the University of Guilan, Iran in spring 2018. Fruit was obtained from all female plants in 2020, and cold storage and sensory evaluations were carried out for three years between 2020 and 2022. Genotypes that passed this evaluation were selected as potential cultivar candidates.

### First stage of genotypes evaluation

Fruits were harvested from uniform vines with the same cultural practices about 160 days after full bloom. During harvesting, special attention was paid to ensuring that the fruit flesh acquired the desired yellow color, which is important for varieties with yellow fruit flesh. Immediately after harvest, the fruits were taken to the postharvest laboratory at University of Guilan. While taking kiwifruit samples, care was taken to ensure they were representative of the genotypes and free from any physical injuries, sunburn, stains, bruises, and harmful pathogen infestations.

Two hundred and one female hybrid genotypes and control cultivar (‘Golden’) evaluated and compared regarding 25 different traits (phenological and pomological traits). The 25 different traits of this study are shown in Table 2. In addition, the frequency values ​​of 7 important traits of 201 hybrid genotypes and controls are shown in Fig. 2. After this preliminary evaluation, second-stage analyses were carried out in the following years with genotypes with fruit weights of 90 g and more.

**Table 2:** Descriptive statistics of characteristics of 201 kiwifruit genotypes and control

Variable	Mean	Minimum	Maximum	Variance	Std. dev.	Coeff. of variation
Fruit weight	70.19	50.46	120.98	233.91	15.29	21.79
Fruit length	58.45	48.61	76.74	45.32	6.73	11.52
Maximum diameter	46.77	38.97	55.36	12.75	3.57	7.64
Minimum diameter	42.58	35.57	53.15	13.00	3.61	8.47
Length - width ratio	1.25	1.02	1.68	0.02	0.14	10.86
Core length	12.26	7.32	24.19	11.63	3.41	27.82
Core width	6.10	4.21	9.85	2.33	1.53	25.02
Dry matter	17.87	15.18	20.47	1.64	1.28	7.16
Hue angel	102.63	92.82	111.93	26.69	5.17	5.03
Flesh firmness	7.11	4.67	9.37	1.32	1.15	16.17
Soluble solids content	10.07	8.23	14.10	1.59	1.26	12.51
Titratable acidty	1.36	0.87	1.69	0.04	0.20	14.54
Brix - acid ratio	7.63	4.89	16.02	3.80	1.95	25.53
Ascorbic acid content	81.52	41.27	155.64	422.81	20.56	25.22
Percentage of budbreak	68.32	51.66	87.18	59.63	7.72	11.30
Time of budbreak	8.62	0.00	21.00	14.84	3.85	44.67
Percentage of flowering shoots	71.61	52.45	88.75	76.14	8.73	12.18
Percentage of vegetative shoots	28.39	11.25	47.55	76.13	8.73	30.74
Number of flowers in winter buds	8.27	2.67	14.00	11.02	3.32	40.14
Number of kingflowers in winter buds	4.85	2.00	6.67	1.12	1.06	21.86
Number of lateral flowers in winter bud	3.42	0.00	8.34	8.49	2.91	85.16
Flowering period	7.02	4.00	10.00	2.03	1.43	20.30
Fruit count	129.48	53.00	405.00	3926.08	62.66	48.39
Percentage of deformed fruits	2.40	0.00	10.79	2.25	1.50	62.64
Yield	8.65	3.26	21.66	12.33	3.51	40.61

All vines of genotypes applied the same pruning practices. The vine’s characteristics, such as the number of buds left after pruning, the number of breaking buds, the number of fertile buds and the number of flowers, were recorded for each vine. From these data were done the following indexes: bud break index (AI) = (number of breaking buds / number of buds left after pruning) × 100; fertility index (FI) = number of flowers / number of fertile buds; real fertility (RF) = number of flowers / number of buds left after pruning [31, 32]. Dimensions of length, longest and shortest diameter, and length and width of core were measured using a digital caliper gauge with a sensitivity of 0.01 mm.

An electronic balance measured the fruit weight to an accuracy of 0.001 g. All 201 female genotypes were screened according to fruit weight, and if fruit weight was under 90 g, they were removed from the experimental orchard. Thirty-one genotypes were selected with 90 g fruit weight after the first evaluation.

### Second stage of genotypes evaluation

According to Plant & Food Research (PFR) standards, hybrid genotypes with fruit weights of 90 g and more and dry matter content (≥ 17%), hue angle (≤ 103°), fruit flesh firmness (≥ 6 kg/cm2), water-soluble solid content (≥ 10° Brix), titratable acids (≥ 0.9%) and ascorbic acid content (≥ 60.82 mg/100 g FW). Twenty-one female genotypes that scored above the threshold were selected for a sensory test (Fig. 2). Fruit quality was analysed according to Nardozza and Burdon [33]. The firmness of fruits (kg) was measured using an Effegi penetrometer (model FT 011, USA) with an 8 mm diameter probe. The probe was placed at two locations on opposing sides of the equatorial plane of the fruit after peeling off a 2 mm layer from the outer surface of the fruit [34]. Kiwifruit juice measured titratable acidity (TA) and soluble solids content (SSC). The SSC value of the samples was determined as a percentage of sugars (%) using a digital pocket refractometer (Euromex RD 635, Netherlands). Measurement of TA represented as the concentration of citric acid (%), involved titrating the fruit juice against 0.1 N NaOH while using phenolphthalein as the pH indicator [35]. The SSC value was divided by the TA value to calculate the BAR. The pH of kiwifruit juice was measured by a digital pH meter (Hanna instrument, model HI 8519, Italy [36]. In this study, the amount of ascorbic acid (vitamin C) in different kiwifruit candidates was determined by using redox titration of vitamin C with 2,6-dichloroindophenol (DCP), following the CNS GB/T6195–1986 protocol [37].

To capture images of the kiwifruit samples, we used an apparatus developed in the Department of Biosystems Engineering of the University of Guilan. The main components of the image-capturing system were an illumination chamber with a lighting system and a 10-megapixel digital camera (Basler acA3800- 10gc, Basler AG, Germany) with a graphical user interface (Basler Pylon viewer application, Version 6.3.0.23157, Basler AG, Germany) to control and adjust the camera. To prepare images of the middle-cut surface of the samples, the kiwifruit samples were cut in the middle along the equatorial plane and placed inside the chamber on a ring with the cut surface facing the camera lens. The samples were placed on matte white cardboard in the illumination chamber, and the camera was positioned 20 cm above the samples. The red-green‐blue (RGB) images with a resolution of 3840 × 2748 pixels were captured and saved.

**Fig. 1:**
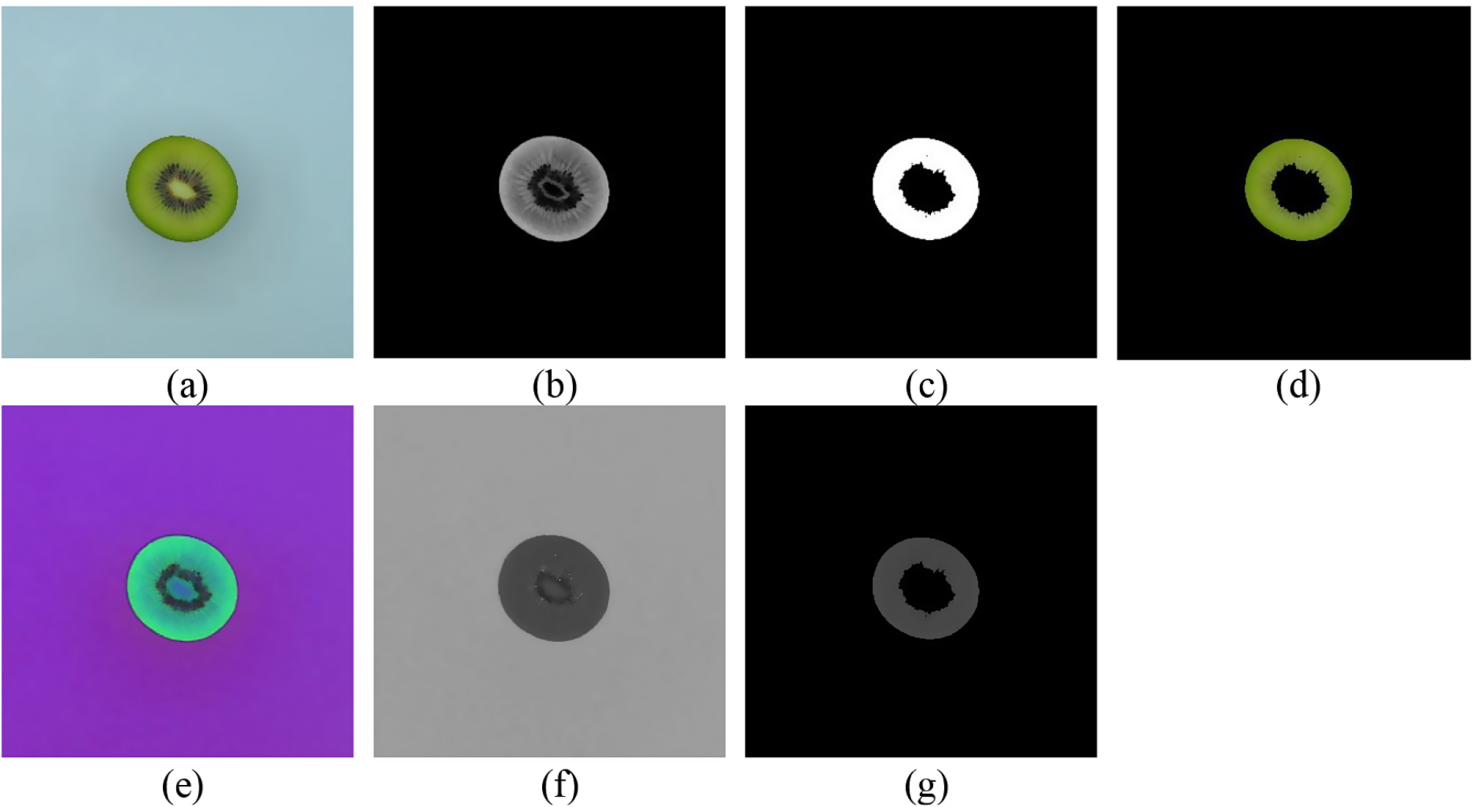
Gallery of middle-cut images at different steps of image processing. (**a**) RGB image of kiwifruit middle cut, (**b**) ExG image, (**c**) binary image of outer pericarp regions, (**d**) RGB image of outer pericarp regions, (**e**) HSV image of kiwifruit middle cut, (**f**) H component of kiwifruit middle cut, and (**g**) H component of kiwifruit outer pericarp regions

**Fig. 2:**
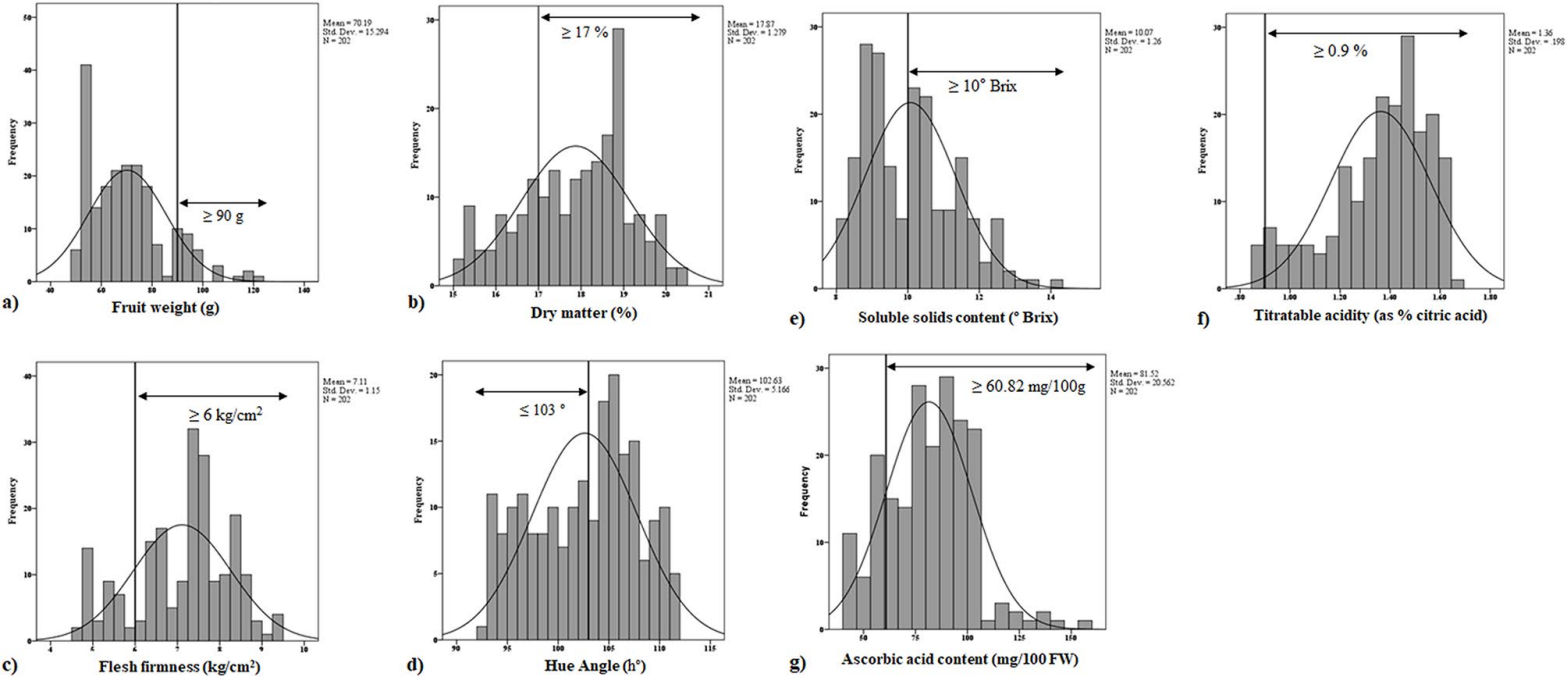
Frequency charts of (**a**) Fruit weight, (**b**) Dry matter, (**c**) Flesh firmness, (**d**) Hue angle, (**e**) Soluble solids content, (**f**) Titratable acidity and (**g**) Ascorbic acid content in 201 genotypes and Golden kiwifruit (control)

In order to accurately determine the flesh color of genotypes, the hue value of the outer pericarp regions was extracted using image processing techniques. The captured images of kiwifruit samples were transferred to the computer and loaded in the image processing toolbox of MATLAB programming software (R2021a, the MathWorks, USA). At first, to reduce the number of calculations, a block of 1600 × 1600 pixels was cropped from the images, which included the kiwifruit middle cut image (Fig. 2a). The Excessive Green (ExG) image was calculated using the formula ExG = 2 * green – red – blue to isolate the outer pericarp regions. After applying optimal thresholding and morphological opening operations to the ExG image, the outer pericarp regions were segmented and separated (Fig. 1c). To provide a clear visual representation for readers, the obtained binary image of the outer pericarp was overlaid with the original RGB image to produce the RGB images of the outer regions (Fig. 1d). Conversely, the RGB image of the fruit’s middle cut was transformed into the HSV colour space (Fig. 1e) to obtain the hue colour layer (Fig. 1f). The hue component image of the outer pericarp regions (Fig. 1g) was generated by overlaying the hue colour layer and the binary image of the outer areas. In this image, the background regions had a value of zero. The average hue value of the outer pericarp regions in this image was then calculated and used for further analysis.

### Third stage of evaluation (sensory evaluation) of genotypes

Sensory evaluation was conducted based on a pilot study on Kiwifruit texture [38]. The fruits collected at harvest time were placed in plastic containers and covered with thin polyethylene, then kept at room temperature (approximately 25 °C) to ripen and become ready for sensory evaluation. Evaluations were conducted using an in-house panel of 12 staff members aged 22 to 55 years, of whom 7 had previous experience in tasting and evaluating kiwifruit. Before the kiwifruit tastings commenced, the panel was familiarised with textural attributes such as mechanical features (soft core, hard core and chewy core), geometrical features (grainy, floury, lumpy, mealy, mushy, starchy, webby, jelly, cellular flesh), mouthfeel (melting, smooth/creamy, gelatinous, slimy, astringent), moisture content (juicy, watery, dry) as well as contribution of seeds to texture (crunchy, gritty, seedy) by tasting texture examples. Each evaluation session was conducted when the fruit reached eating-ripe firmness, with available participants that day. Participants assessed each fruit according to a series of predetermined texture attributes. If a texture attribute was present, a tick was placed in the box provided on the provided ballot sheet. They also commented on whether they considered the texture novel and, if so, positive or negative [38]. In the final stage, only 13 genotypes were selected due to sensory testing, as they were found acceptable by consumers with their unique aroma and taste.

### Statistical analysis

In this study, we utilized descriptive statistical analysis, focusing on frequency distribution charts. This method was chosen to effectively summarize and visually represent the collection data, making it easier to identify patterns and trends. The primary purpose of a frequency distribution patterns and determine appropriate range of numeric values. Pearson correlation was used to measure the degree of relationship between two variables. Principal component analysis (PCA) biplot was used to depict the relationship between the estimated variables and the hybrid genotypes. Descriptive statistics were performed using SAS software [39] (version 9.4, SAS Institute Inc., Cary, NC, USA). Quantitative analysis of traits and graphs was performed using SPSS version 25 and Origin 2022 [40] software.

## Results

### Genetic diversity analysis based on fruit and vine characteristics

The results of variation analysis of primary fruit morphological and pomological (quality characteristics) from 201 kiwifruit genotypes and control are summarised in Table 2. Among the 25 studied characters, the variation coefficient ranged from 5.03 to 85.16%, with an average of 25.49%. The number of lateral flowers in winter buds showed the most significant variation coefficient, followed by the percentage of deformed fruits (62.64%), fruit count (48.39%), time of bud break (44.67%), yield (40.61%) and number of flowers in winter buds (40.14%). The lowest variation coefficient of 5.03%, was found in the fruit flesh Hue angle.

The fruit weight ranged from 50.46 to 120.98 g, with an average weight of 70.19 g. Fruit length ranged from 48.61 to 76.74 mm, the maximum diameter 38.97 ∼ 55.36 mm and the minimum diameter 35.57 ∼ 53.15 mm, length-width ratio (LWR) 1.02 ∼ 1.68, core length 7.32 ∼ 24.19 mm and core width 4.21 ∼ 9.85 mm (Fig. 2a).

Dry matter percentage ranged from 15.18 to 20.47%, averaging 17.87%. In the present study, the hue angle ranged from 92.82° to 111.93°, with an average of 102.63°. Flesh firmness ranged from 4.67 to 9.37 kg/cm^2^, averaging 7.11 kg/cm^2^. The soluble solids content ranged from 8.23 to 14.10° brix, with an average of 10.07° brix. Titratable acidity ranged from 0.87 to 1.69%, averaging 1.36%. The brix-acid ratio ranged from 4.89 to 16.02. Ascorbic acid content ranged from 41.27 to 155.64 mg/100 g FW, averaging 81.52 mg/100 g FW.

The percentage of bud break ranged from 51.66 to 87.18%, with an average of 68.32%, and the time of bud break ranged from 0 (same as control) to 21 days after control. The percentage of flowering shoots ranged from 52.45 to 88.75%, the percentage of vegetative shoots ranged from 11.25 to 47.55%, the number of flowers in winter buds ranged from 2.67 to 14.00, the number of king flowers in winter buds ranged from 2 to 6.67, and the number of lateral flowers in winter bud ranged from 0 to 8.34. Kiwifruit typically produces an inflorescence with a terminal flower and two lateral flowers. However, the lateral flowers mostly fail to develop fully and often abort before blooming [41].

As a result of the studies carried out to extract the colour tone from the pericarp cross-section of kiwifruit genotypes, it was clear that there was a significant difference in the colour intensity between the outer pericarp areas in the fruit middle-cut image and the image background regions in their greenness (Fig. 1).

According to New Zealand’s Plant & Food Research standards, to select genotypes with superior traits, they must have a minimum dry matter content of 17% [42]. As shown in Fig. 2b, the dry matter distribution is roughly bell-shaped and appears to have a normal distribution with a central peak and a symmetrical data spread on both sides. This indicates a fairly consistent dry matter content in the sample. The data obtained are within one standard deviation of the mean (approximately 16.5 and 19.25). A smaller standard deviation means the data points are clustered more closely around the mean, indicating relatively consistent dry matter levels in the sample. Of the 201 genotypes evaluated, 147 exceeded 17% dry matter. The control cultivar recorded a dry matter of 18.34%.

As shown in Fig. 2c, the distribution appears roughly bell-shaped, indicating a normal distribution with a central peak and a symmetrical spread of data on either side. The data is centred around a mean of 7.11 and has a standard deviation of 1.15. Most observations fall within one standard deviation of the mean (between approximately 5.96 and 8.26). The data is clustered around the mean, with a gradual decrease in frequency as we move away from the centre. The normal curve provides a good visual representation of the data’s distribution. Among the 201 genotypes evaluated, 164 exhibited flesh firmness greater than 6 kg/cm².

According to Fig. 2d, the distribution of hue angle is roughly bell-shaped distribution, suggesting a normal distribution with a central peak and a symmetrical spread of data on either side. The average hue angle for the kiwifruit sample is 102.63°. From all the evaluated genotypes, 96 genotypes showed a hue angle of less than 103 degrees. In contrast, the control cultivar’s hue angle was measured at 109.94 degrees, indicating a greenish-yellow flesh colour in this cultivar. Most observations fall within one standard deviation of the mean (between approximately 97.46° and 107.80°), based on the provided standard deviation of 5.166. It offers valuable information for understanding the consistency and variability in colour, which can influence consumer appeal, processing methods, and overall quality control.

As shown in Fig. 2e, the histogram of SSC resembles a bell-shaped curve, indicating a normal distribution with a central peak and a symmetrical data distribution on both sides. This indicates a fairly consistent SSC across the genotypes. The data is centred around a mean of 10.07° Brix and has a standard deviation of 1.26. The majority of kiwifruit in this value have an SSC close to the average of 10.07, indicating a relatively consistent level of sweetness.

As shown in Fig. 2f, the distribution of titratable acidity is approximately normal, suggesting a normal distribution with a central peak and a symmetrical spread of data on either side. This indicates a fairly consistent titratable acid content across the genotypes. The data is centred around a mean of 10.07 and has a standard deviation of 1.26. The peak of the histogram, representing the most frequent titratable acid value, appears to be between 1.4 and 1.5%. Among the 201 genotypes evaluated, the titratable acidity value of 196 was determined to be above 0.9%.

According to Fig. 2g, the distribution of ascorbic acid content is slightly skewed to the right, meaning there are more kiwifruit with lower ascorbic acid content than higher content. The data is centred around a mean of 91.52 and has a standard deviation of 20.562. While the data suggests a general tendency towards a normal distribution, the relatively high standard deviation points to some variation in ascorbic acid content within the sample.

**Fig. 3:**
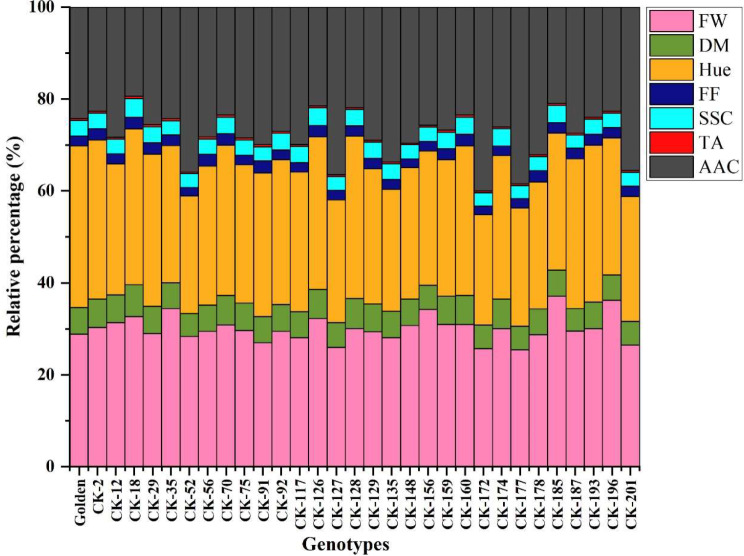
The changes of fresh weight (FW), dry matter (DM), Hue angle (Hue), flesh firmness (FF), soluble solid content (SSC), titratable acidity (TA), and ascorbic acid content (AAC) in 30 selected genotypes and control cultivar (‘Golden’)

Figure 3 shows the results of seven superior fruit quality parameters (FW, DM, Hue, FF, SSC, TA, AAC) in the 30 genotypes which passed to the second stage of screening and control. Each bar represents a genotype, and the different coloured sections within each bar show the contribution of each trait or component (FW, DM, Hue, etc.) to the overall makeup of that genotype. The bar chart provides a good overview of the compositional differences between different kiwifruit genotypes. We found that the relative percentage of each characteristic varied significantly between genotypes.

The highest FW values were found in genotype CK-185 and the lowest ones in CK-29. Genotypes CK-128 and CK-187 have the highest and lowest values of DM respectively. Genotypes CK-172 showed the highest fruit flesh hue angle and the lowest value was found in CK-128 it is the highest. Genotype CK-91 showed the highest FF and the lowest value was found in genotype CK-174. In genotypes CK-185 and CK-187, the SSC has the highest and lowest values, respectively. With genotype CK-178, the TA trait has the highest value, while with genotype CK-12, it has the lowest value. The AAC has the highest value in genotype CK-172 and the lowest value in genotype CK-18.

### Correlation analysis on the main characters

**Fig. 4:**
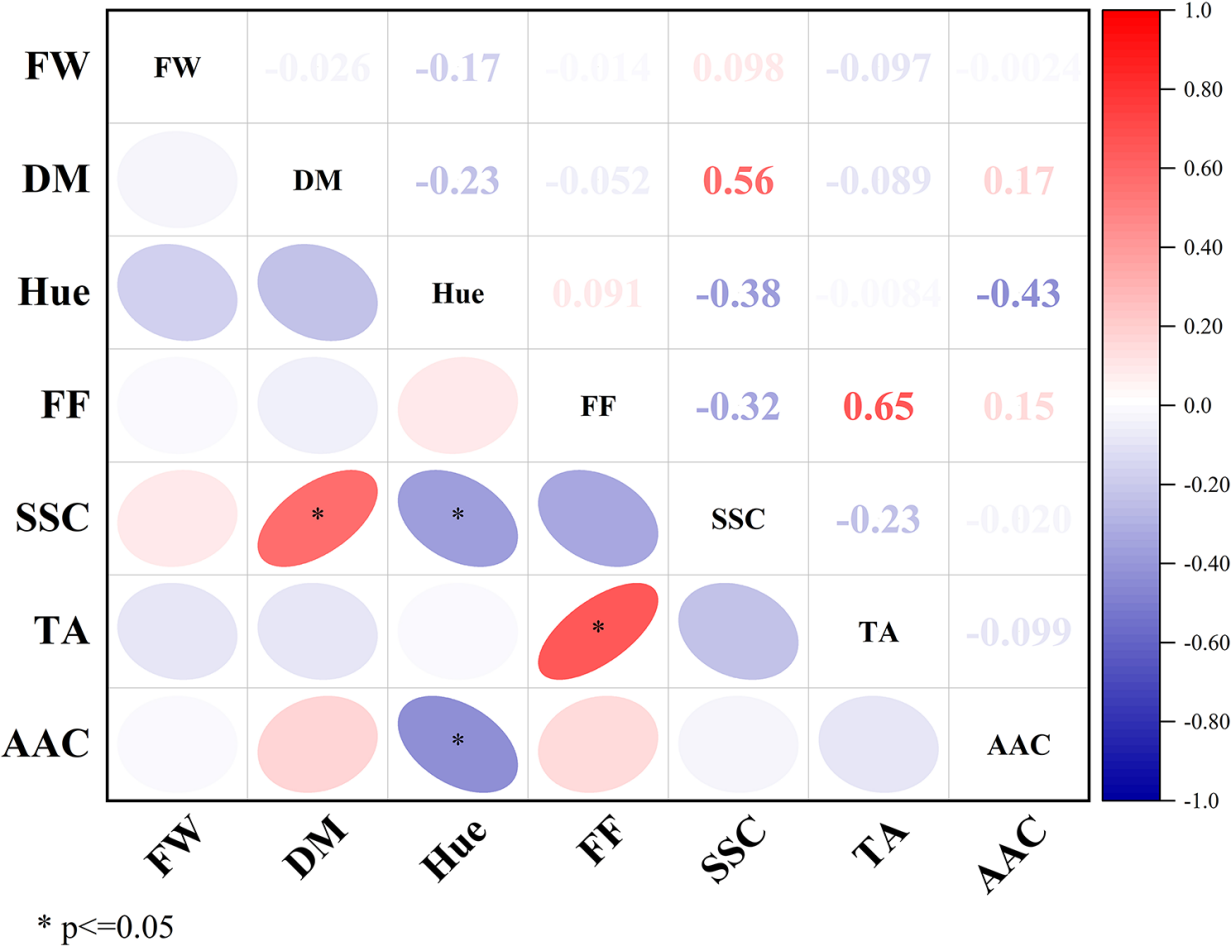
Pearson correlation of seven variables depicted with a heatmap (red indicates positive whereas blue indicates negative correlation) in 30 selected genotypes and control cultivar (‘Golden’)

Pearson correlation was used to measure the degree of relationship between two variables. The results showed that there is a positive correlation between FF and TA (R2 = 0.65). This means that the firmer fruits tend to be the more acidic, which is a critical factor for determining flavor profiles (Fig. 4). In addition, a significant positive correlation was observed between SSC and DM (R2 = 0.56), which suggesting that the sweeter fruits had the higher dry matter content, contributing to their enhanced overall quality.

### **Biplot analysis**

**Fig. 5:**
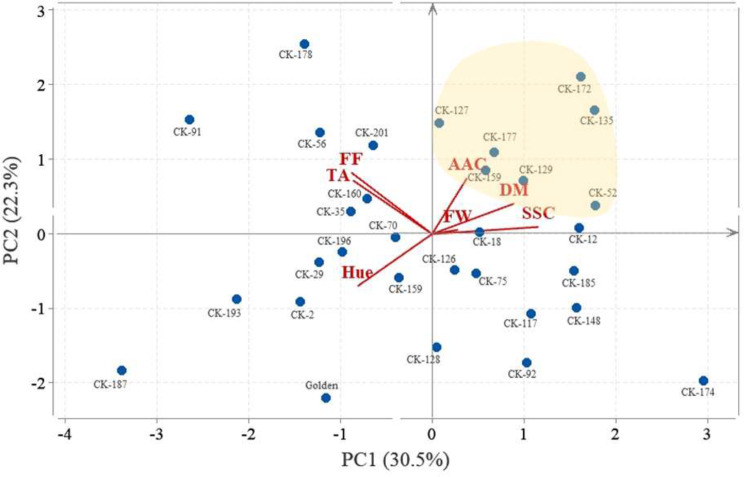
Correlation heat map of fresh weight (FW), dry matter (DM), Hue angle (Hue), flesh firmness (FF), soluble solid content (SSC), titratable acidity (TA), and ascorbic acid content (AAC) in 30 selected genotypes and control cultivar (‘Golden’)

Principal component analysis (PCA) biplot depicting the relationship between the estimated variables and the hybrid genotypes (Fig. 5). PC1 explains 30.5% of the variation in the data, while PC2 explains 22.3%. This means that these two components capture a significant portion of the variability in fruit quality. The arrows representing the variables (FW, AAC, DM, SSC, Hue, FF, TA) show their direction and magnitude of influence on the principal components. Variables pointing in the same direction are positively correlated. For example, DM and SSC are positively correlated. Longer arrows indicate a stronger influence of that variable on the principal components. Some genotypes might be far from the main cluster, indicating that they have unique combinations of quality attributes. This biplot helps us identify the genotypes with potential for high SSC, DM and AAC including CK-52, CK-129, CK-159, CK-177, CK-127, CK-135 and CK-172.

### Sensory evaluations

**Fig. 6:**
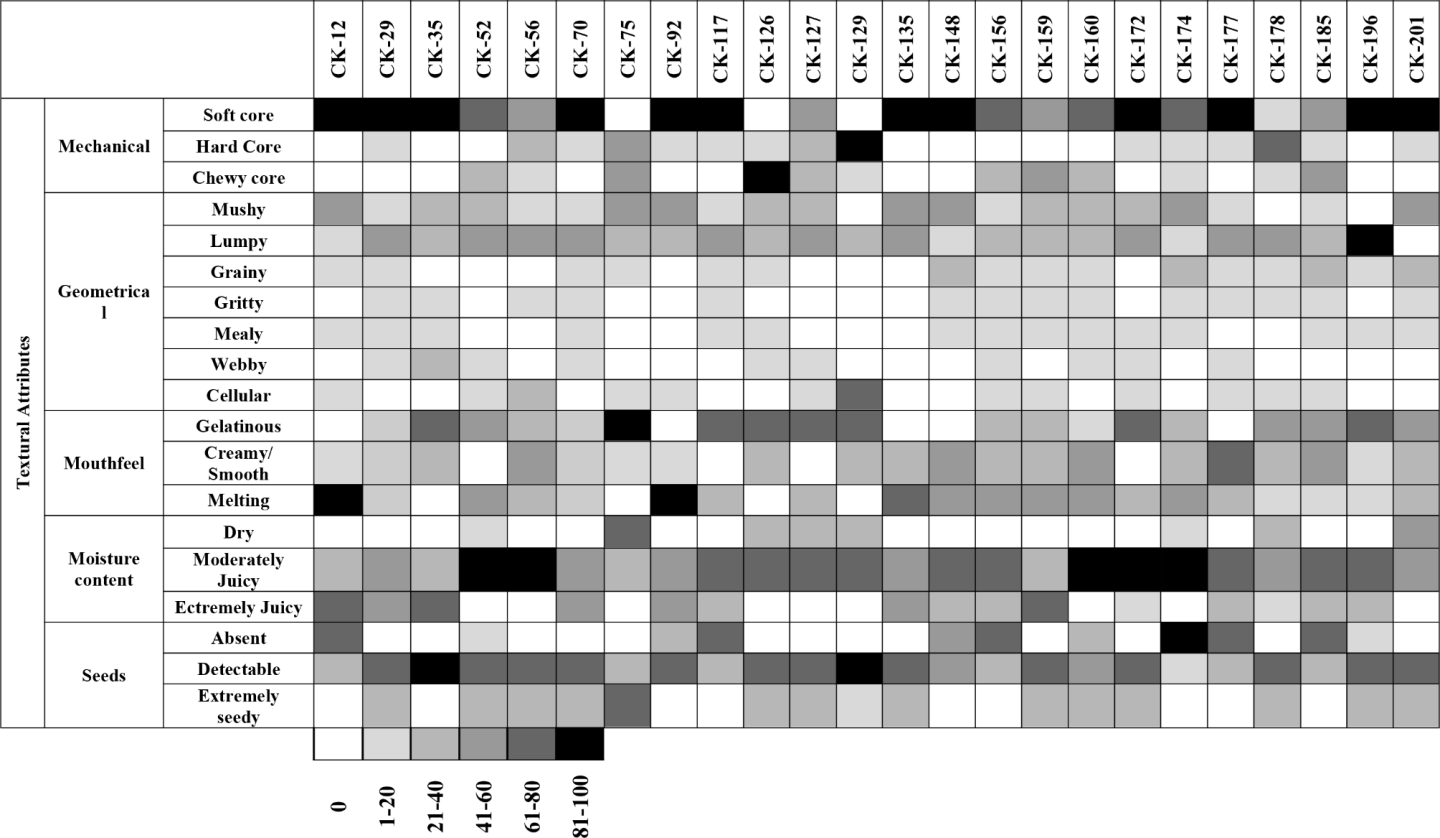
Proportion of all participants’ (*n* = 12) texture scores for 24 kiwifruit genotypes shown as a heatmap, with colours indicating the proportion of participants that scored the fruit for the different texture attribut

The results showed that from 24 studied genotypes, six genotypes showed soft-core fruits and four genotypes showed hard or chewy cores. Most of the genotypes produced fruits with mushy and lumpy geometrical attributes. However, fruits with grainy, gritty, mealy, webby or cellular core textural attributes were also present but not common. Two genotypes induced a melting sensation in the mouth but only one genotype showed a gelatinous mouthfeel. According to more than half of the panel members, the fruit from one genotype, CK-75, had a dry mouthfeel, whereas most other genotypes were found to be moderately juicy. In most genotypes, seeds were detectable. Most of the panel members found one genotype to have non-detectable seeds, while in two genotypes, most participants found the fruit extremely seedy. The results also showed that just 12 genotypes from 24 genotypes showed a ‘novel texture’ by at least half of the participants (Fig. 6).

Upon rating whether these novel fruit textures observed were positive or negative, only one genotype was described unanimously by all participants as having only positive textures (Fig. 6). The fruit of this genotype was described as resembling a jelly-like lollipop; it was moderately juicy with a soft core, gelatinous, homogeneous flesh texture, and undetectable seeds. The texture of fruit of another genotype was also rated positively, but only by half the panel, and only just over half the panel deemed it a novel texture. Five other genotypes with novel textures were mainly positively rated, with a few negative textural characteristics (Fig. 6). They were moderately to extremely juicy, with a degree of lumpiness and detectable seeds (Fig. 6).

One was gelatinous, creamy/smooth with a somewhat gritty flesh texture and a hard core that, for some panellists, made it difficult to scoop. Another had a small but chewy core and somewhat creamy/smooth and melting texture. Several participants noted that the flesh was like a very firm mango or pear, was homogeneous and, on chewing, the flesh easily broke down like a firm but juicy agar jelly. The flesh was described as almost crunchy but smooth and very juicy. Yet another had a soft core with a creamy/smooth and melting texture, which several participants compared to a banana. The novel textures in four genotypes were equally positive and negative, whilst in four the negative textures outweighed the positive ones. All the four genotypes that displayed predominantly negative novel textures had detectable seeds. One of these was described by most of the participants as having an entirely negative novel texture: a soft or chewy core, lumpy and gelatinous flesh and dry to moderate juiciness. Some participants also noted it had a mandarin-like texture with large seeds, tough skin and was astringent (Fig. 6).

**Fig. 7:**
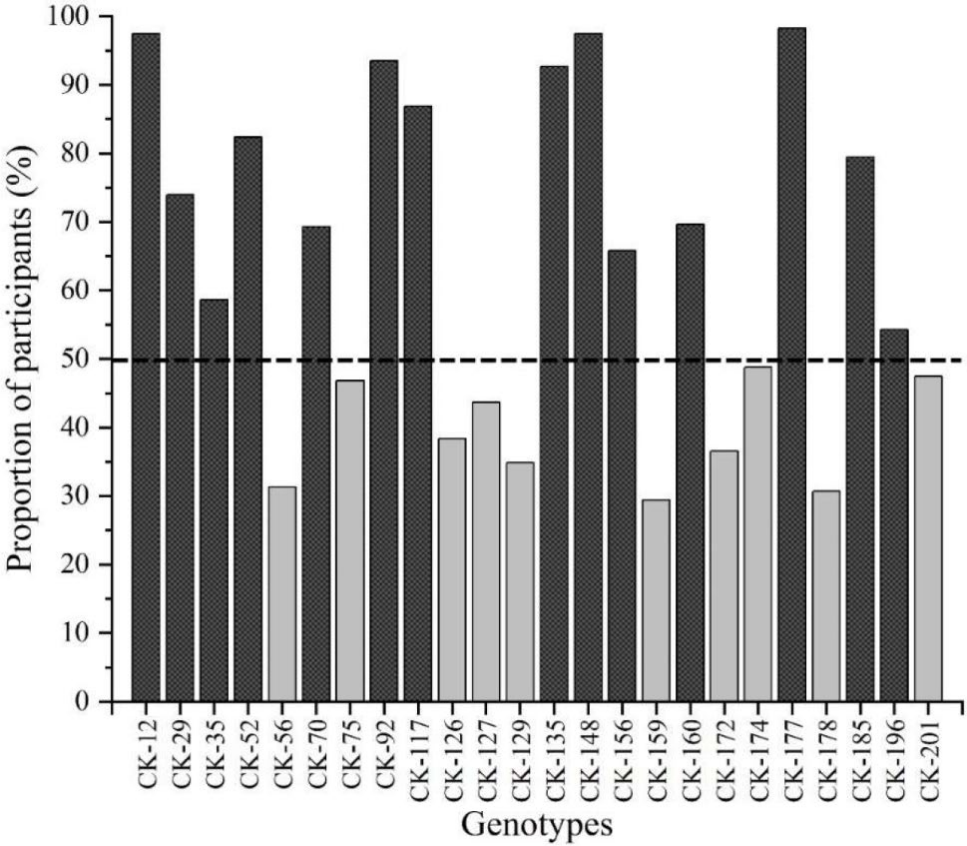
The proportion of experienced panel participants scoring textures of 24 kiwifruit genotypes as novel characteristics. (The horizontal line shows that at least 50% of participants confirmed novelty in texture)

**Fig. 8:**
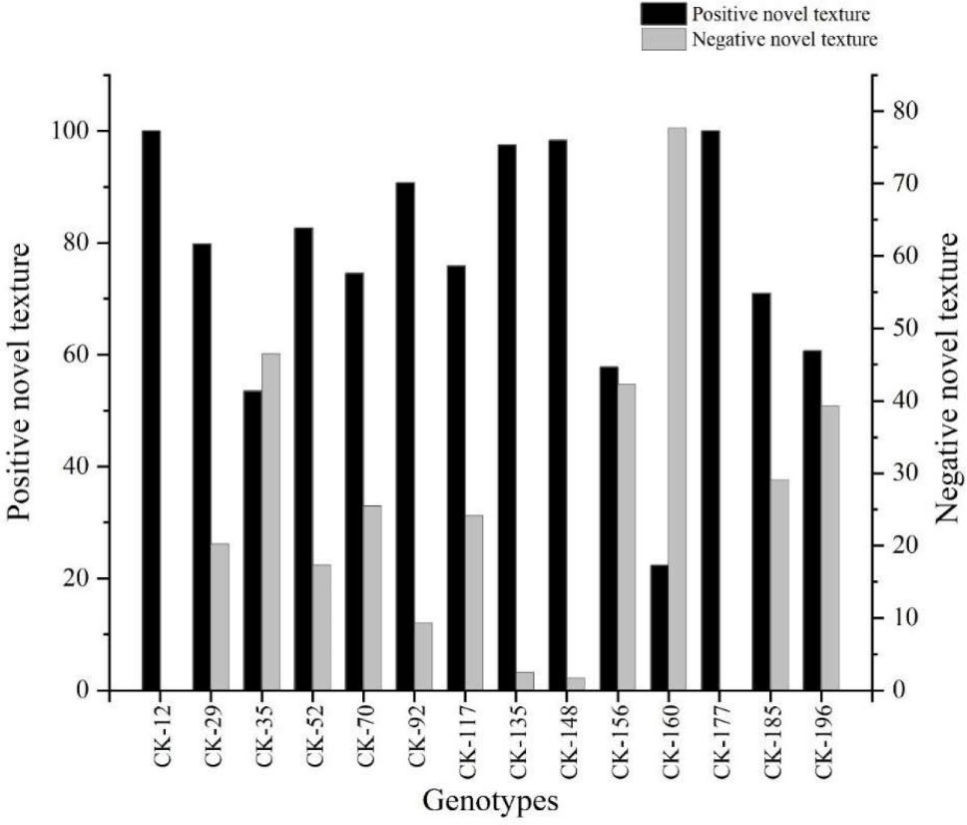
The proportion of experienced panel participants (12) scoring positive and/or negative texture attributes of the selected kiwifruit genotypes

The results also showed that only 14 genotypes from 24 genotypes have a ‘novel texture’ by at least half of the participants (Fig. 7). Upon rating whether these novel fruit textures were positive or negative, only two genotypes (CK-12 and CK-177) were described by all participants have 100% novelty with positive texture (Fig. 8). Fruits of these two genotypes were described as being moderate to extremely juicy with a soft core, melting or creamy homogeneous flesh texture (Fig. 6).

The texture of fruit from 3 other genotypes (CK-92, CK-135 and CK-148) also showed positively by more than 90% of the panel members. Eight other genotypes with novel textures were mainly positively rated, with a few negative textural characters. One genotype (CK-160) was considered to have negative texture according to the majority of panel members (Fig. 8). Finally, 13 genotypes showed a positive texture by more than 50% of panel members and selected for the final breeding stage.

## Discussion

In this study, the phenological, pomological and sensory characteristics of different hybrid genotypes was compared with golden kiwifruit grown commercially in Iran. We found that some hybrid genotypes produced lateral flowers and the others produced just king flower and their lateral flowers normally aborted before blooming. There was also a significant difference in the flowering period between hybrid genotypes, ranging from 4 to 10 days. The fruit count ranged from 53 to 405. The percentage of misshape fruits ranged from 0 to 10.9%. Previous studies also showed higher misshape fruit is related to the higher crop load [43]. However, the percentage of deformed fruit formation may vary depending on the region and year, and this number tends to increase in strongly developing shoots [44]. The results showed that genotypes with cymose inflorescences produced more fruits, which resulted fruit weight decline less than 90 g. Fruit yield varied between 3.26 and 21.66 kg and was determined as 8.65 kg on average. However, this situation shows that the increase in yield in these genotypes is due to a higher quantity of smaller fruits. Prevous study by Antognozzi et al. [45] showed that lateral flowers consistently produce smaller fruits, regardless of the number of flower buds on the shoot. This is due to the reduced vascular tissue in these flowers compared to the terminal flower. Similar observations have been made in pomegranates, where terminal flowers yield larger fruits than lateral ones [46]. Also, in grapes, asynchronous cell division has been reported to cause changes in fruit size in over-branched inflorescences [47]. Richardson et al. [41] stated that in kiwifruit, the delayed development of lateral flowers compared to the terminal flower results in smaller ovaries and smaller lateral fruits. In contrast, genotypes with single-flower inflorescences produced bigger fruits. Richardson et al. [41] reported that early blooming of terminal flowers in kiwifruit have larger ovaries, which correlate positively with larger fruit size. In a kiwifruit breeding program in Turkey, large, hairless and yellow-fleshed genotypes were first aimed to develop. Researchers selected hybrid genotypes larger than 80 g as potential cultivar candidates. They monitored seedlings from crosses of *A. chinensis* ‘Jintao’, *A. chinensis* ‘Hort16A’, *A. chinensis* type 17,684, and *A. deliciosa* ‘Hayward’ × ‘Tomuri’ over three years for their phenological and pomological traits. Of the 133 seedlings planted, three genotypes were selected as superior cultivars due to their large fruits with minimal hair; two of these had better aromatic and flavour profiles, and one had a red circle around the fruit core [48]. Taking into account the market preference for fruits of 90 g and above, we primarily selected genotypes that weigh 90 g and above under natural growing conditions without the need for any chemical application.

The fruit dry matter (DM) is potentially another useful taste indicator for kiwifruit. It is reasonably constant during ripening with only small losses due to respiration. The knowledge that kiwifruit dry matter content has a positive influence on consumer taste preference; most consumers prefer higher dry matter kiwifruit. Dry matter percentage of different haybris genotypes ranged from 15.18 to 20.47%. Lv et al. [49] found that the dry matter ratios of 66 kiwifruit genotypes ranged from 14.30 to 19.80%. In breeding studies, priority is given to varieties with higher firmness in terms of storage life [50]. In our research, we selected hybrid genotypes with fruit flesh firmness of 6.9 kg and more.

Plant & Food Research (PFR) Institute in New Zealand standards suggest that a minimum soluble solids content of 10° Brix at harvest is required for selecting promising genotypes. Additionally, Burdon et al. [51] stated that soluble solids content in kiwifruit is indicative of its eating quality, with fruits harvested at a higher Brix degree having better market appeal. Hundred one of the hybrid genotypes have this recommended total soluble solids value of 10° Brix. Lv et al. [49] reported that they determined a titratable acidity range between 0.98% and 1.76% in 66 different kiwifruit genotypes in their study. The vast majority of our genotypes had acidity values above 0.9. The researchers reported that total organic acids in kiwifruit decreased during harvest, ripening and storage and added that the amount of titratable acidity may vary depending on the cultivar and season, which is consistent with the results of this study [52, 53].

Kiwifruit is known especially for its high vitamin C content (ascorbic acid). It is also reported that yellow-fleshed varieties have a higher vitamin C content [54]. In our previous studies, we determined the threshold ascorbic acid content as 60.82 mg/100 g FW in the ‘Hayward’ cultivar grown in the Guilan region [55]. We detected ascorbic acid content above this threshold in only 30 of the 201 genotypes evaluated. In the control cultivar, an ascorbic acid content of 75.91 mg/100 g FW was recorded. Changes in vitamin C amounts in fruits, including kiwi, are primarily due to cultivar, growing region and conditions, fertiliser use, harvest maturity, harvest time, storage and ripening conditions [54, 56]. A comprehensive study conducted in China with genotypes belonging to different kiwi species showed that ascorbic acid content increased up to three times (43.75 to 146.86 mg/100 g FW) has been reported to differ. Similarly, it has been revealed that there is a 3-fold difference between the hybrid genotypes in our study.

The results showed a significant difference in key fruit quality parameters between kiwifruit genotypes, which is important for breeding programs. The fruit weight (FW), dry matter (DM), hue, firmness (FF), soluble solids content (SSC), titratable acidity (TA), and ascorbic acid content (AAC) are the main characteristics that impact consumer liking and fruit marketability. The variation in fruit weight (FW) across genotypes is a key indicator of their overall size, better potential yield and consumer preference [57]. Genotypes with higher FW (e.g., CK-185, CK-196 and CK-35) could be preferable for kiwifruit growers. A higher percentage of dry matter is associated with increased taste and consumer preference [58]. Hue angle can influence market appeal; FF is important for consumer satisfaction. SSC indicates superior sweetness, and TA contributes to flavour balance [58]. Consumer preference for kiwifruit requires a balance between sweetness and acidity, which is crucial for its flavour profile, and SSC and TA are key determinants of taste quality [59]. However, Genotypes with high AAC, such as CK-56, CK-91, and CK-174, are important for nutritional value and health properties [54]. The increasing consumer demand for health-promoting foods positions kiwifruit with high AAC as a compelling product in the health food market, appealing to those seeking natural sources of antioxidants and vitamins [60–62]. Growers and breeders can use this data to select superior genotypes with desired traits like higher DM for better flavour, higher SSC for sweetness, higher AAC for nutritional value or specific Hue angle for market appeal. Understanding these compositional variations can inform breeding strategies to develop new kiwifruit varieties with enhanced qualities, potentially leading to increased profitability and market competitiveness [63].

Research over the past decade has examined consumers’ attitudes, beliefs and perceptions towards fruits in general, as well as the effects of taste, novelty and appearance on kiwifruit preferences and willingness to purchase. Consumer preference mapping was used to find opportunities for new flavours, and sensory panels were used to create flavour profiles for different genotypes [9]. Texture plays a vital role in the eating experience, with firmness correlating positively with consumer acceptance. A desirable texture is often linked to higher SSC and lower acidity [64]. In contrast, while sensory attributes are critical, some consumers may prioritise nutritional content over sensory experience. Consumers are becoming more health-conscious, often prioritising the nutritional profile of fruits. This can sometimes overshadow sensory enjoyment, indicating a complex relationship between health benefits and sensory enjoyment in fruit consumption [61].

In general, the panellist comments and descriptions of the fruit with novel textures matched their scoring of the texture attribute terms. This could mean that: (a) panellists were unable to describe the novel textures and therefore resorted to using the list of known texture terms, or (b) the novelty of the texture of the fruit was attributed to a combination of known textures being dominant, thus giving the overall impression of being unique. At this point, it is worth noting that a score of ‘novel texture’ was based on each panellist’s personal opinion and, as such, can be viewed merely as an indication of the potential of a kiwifruit cultivar for further, more robust sensory studies. Most participants liked the milder flavour of some genotypes compared with the ‘Golden’ cultivar because it has a “different taste”.

The current study’s findings showed that only 14 genotypes of 24 genotypes have a ‘novel texture’ by at least half of the participants (Fig. 7). A further study on novelty showed that only two genotypes (CK-12 and CK-177) were described by all participants as having a 100% of novelty with positive texture. They produce moderate to extremely juicy fruits with a soft core, melting or creamy homogeneous flesh texture. The rest of the genotypes with novelty showed different ranges of negative and positive textures. One genotype (CK-160) was considered to have negative texture according to the majority of panel members (Fig. 8). In parallel with the results of our study, it is reported that hybrid genotypes using different kiwifruit species, especially *A. chinensis* varieties, are more appreciated as a result of the evaluations of panellists [65]. Finally, 13 genotypes showed a positive texture by more than 50% of panel members and were selected for the next breeding stage.

## Conclusion

Breeding programs for kiwifruit are becoming increasingly crucial in the face of changing consumer preferences and evolving environmental conditions. To meet these demands, breeders select the best ones from the hybrid population they create and transfer them to growers. In this study, the performance of 201 hybrid genotypes was evaluated to develop new golden kiwifruit cultivars with novel flesh colour and flavour and higher consumer liking. The results showed that 13 selected hybrid genotypes including CK-12, CK-29, CK-35, CK-52, CK-70, CK-92, CK-117, CK-135, CK-148, CK-156, CK-177, CK-185 and CK-196 could stand out due to their exceptional fruit characteristics, making them as promising candidates for commercial production. A further study on novelty showed that only two genotypes (CK-12 and CK-177) were described by all participants as having a 100% of novelty with positive texture. In future studies, it is necessary to focus on developing new varieties that are tolerant or resistant to different stress conditions. Because increasing stress conditions with climate change make the job of growers even more difficult. Bringing new varieties into production that will help them and make their work easier will be among the most critical issues of the coming years. Additionally, necessitate a focus on developing a lower chilling requirement varieties. Moving forward, breeding efforts must prioritize these characteristics to ensure the resilience and sustainability of kiwifruit production in warmer climates, where chilling hours are no longer reliably met.

## Electronic supplementary material

Below is the link to the electronic supplementary material.

Supplementary Material 1

## Data Availability

The data that support the findings of this study are available from the corresponding author upon reasonable request.
