# Supplementary figures and images for: Breeding of new kiwifruit (Actinidia chinensis) cultivars with yellow (golden) fleshed and superior characteristics
Source: BMC Plant Biol. 2024 Nov 5;24:1045. doi: 10.1186/s12870-024-05768-0 (PMC11536603; doi:10.1186/s12870-024-05768-0)

**Extra Figure 1.** Selected kiwifruit genotypes


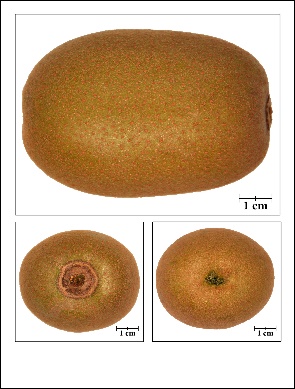

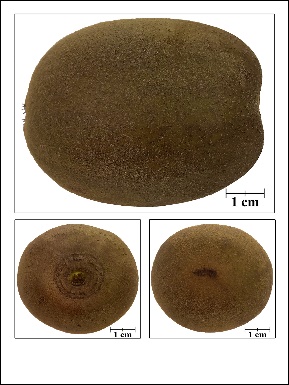

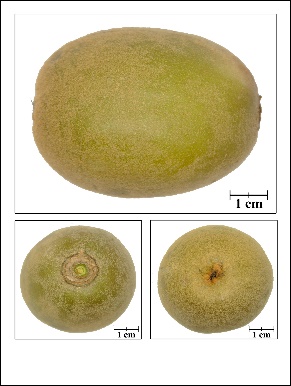

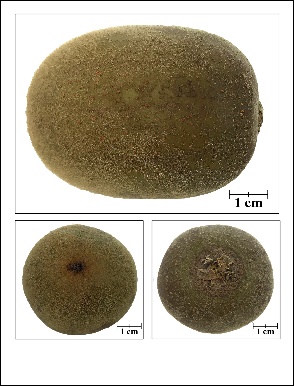

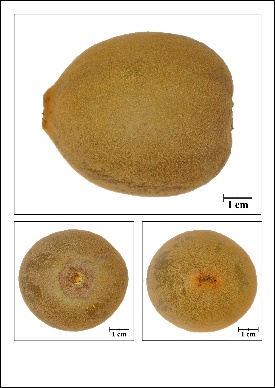

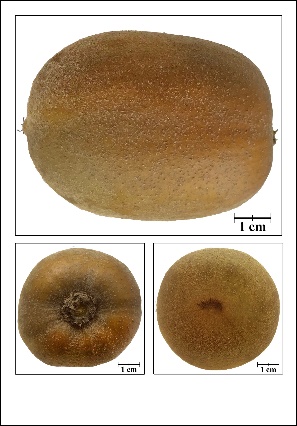

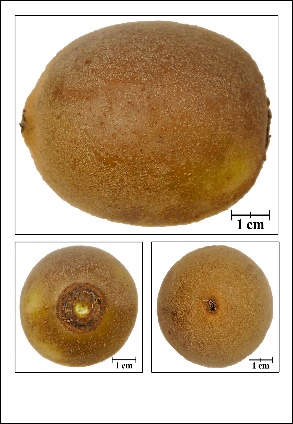

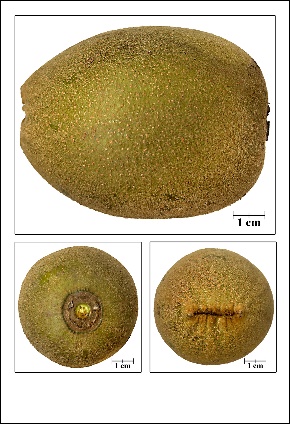

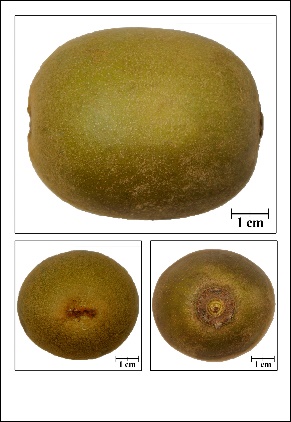

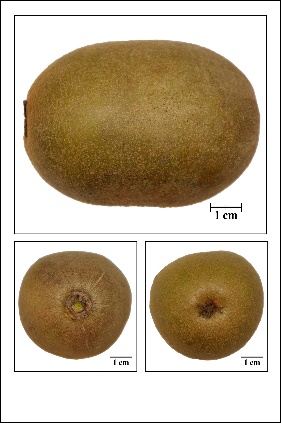

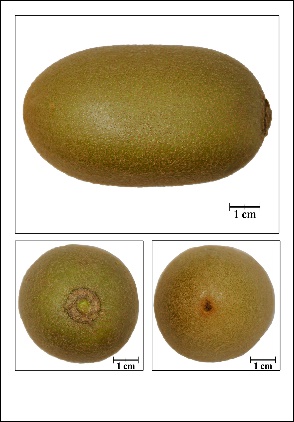

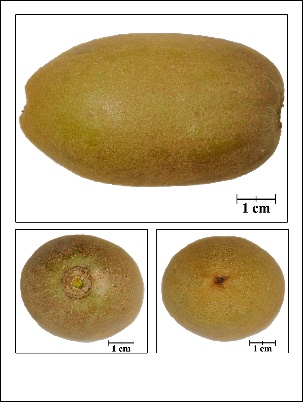

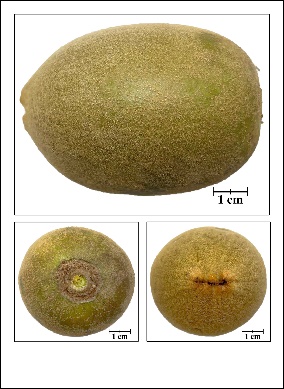

Supplement: Supplementary file 1 — Supplementary Material 1 [file 12870_2024_5768_MOESM1_ESM.docx]
